# Effectiveness of* Citrus* Fruits on* Helicobacter pylori*

**DOI:** 10.1155/2017/8379262

**Published:** 2017-03-20

**Authors:** Giuseppina Mandalari, Carlo Bisignano, Santa Cirmi, Michele Navarra

**Affiliations:** Department of Chemical, Biological, Pharmaceutical and Environmental Sciences, University of Messina, 98168 Messina, Italy

## Abstract

It is known that* Helicobacter pylori* infection is associated with chronic gastritis, peptic ulcer, and gastric carcinoma. Due to the increased side effects of the treatment regimens and the development of antimicrobial resistance, a number of natural compounds have been tested as potential alternatives. In this review, we will examine the current knowledge on the effect of* Citrus* fruits and their derivatives against* H. pylori*, highlighting the remaining outstanding questions on the development of novel therapeutic strategies.

## 1. Introduction

Natural medicines have become increasingly popular for the treatment of certain diseases and occasionally represent the basic ingredients used in medicinal industries. A wide variety of compounds with health promoting properties have been identified in plants. Fruits and vegetables are known to contain at least several hundred different types of phenolic compounds, with identified antioxidant, anti-inflammatory, and antimicrobial properties [1–3]. The antimicrobial properties of natural compounds have gained growing attention over the last few decades: this is due to the increased antibiotic resistance to a vast number of bacterial strains. Therefore, discovering novel bioactive compounds from plant products could help eradicate a number of infectious diseases. We have previously reported the antibacterial activity of polyphenols-rich natural products, including almonds [4, 5], pistachios [6],* Citrus* plants [7],* Vitis vinifera* L. [8],* Olea europaea* L. [9], and* Citrus bergamia* essential oil [10] and juice [11]. In particular, the identification of novel compounds with bactericidal rather than bacteriostatic effect has attracted a lot of interest in recent years. Emerging research areas have also evaluated the role played by polyphenols that reach the large bowel, beneficially modulating the gut microbial ecosystem by increasing the number of* Bifidobacterium* spp.,* Lactobacillus* spp., and* Enterococcus* spp. which are known for their anti-inflammatory, immunoregulatory and cholesterol lowering properties through production of short chain fatty acids [12, 13].

In this review, we examine the current literature on the effect of* Citrus* fruits and their derivatives against* Helicobacter pylori*, a pathogen colonizing the human stomach with high morbidity rate [14]. Due to the increasing emergence of antibiotic resistance to* H. pylori* strains, bioactive compounds present in medicinal plants could represent valuable sources of anti-*H. pylori* agents. Furthermore, we will address the socioeconomic impact of the use of natural compounds in low-income countries with high infection rates of* H. pylori*. Still open questions on the use of novel therapeutic drugs will be highlighted.

## 2. *Citrus*

The term “*Citrus*” refers to a genus of flowering plants belonging to the Rutaceae family. A number of studies report that* Citrus *originated from Southeast Asia, bordered by Northeast India, Burma, and Yunnan (China) [15–17]. Since ancient times,* Citrus* fruits have been widely consumed as important fruit tree crops. Initially, the genus* Citrus* only consisted of a few species, whereas a more extensive list of hybrids and cultivars is available nowadays, mainly based on* Citrus maxima*,* Citrus medica,* and* Citrus reticulata*. The genera* Poncirus* and* Fortunella* are related to* Citrus* spp. According to UN 2007 data, Brazil, China, USA, Mexico, India, and Spain are the largest* Citrus*-producing countries, with commercial* Citrus* growing areas including southern China, the Mediterranean Basin, South Africa, Australia, USA (mainly Florida, California, Arizona, and Texas), Mexico, and parts of South America [17]. The most known examples of* Citrus *fruits, which are widely used for their ornamental, culinary, and medical purposes, are represented by oranges, lemons, grapefruits, and limes. Several studies have highlighted the health promoting properties of* Citrus* fruits, mainly related to the high content in bioactive compounds [18–22].* Citrus* fruit juices could be useful for the prevention of kidney stone formation [23], whereas grapefruits have been proven to lower blood pressure and interfere with calcium channel blockers [24]. Moreover,* Citrus *fruit juices and their flavonoids have anti-inflammatory [18, 25, 26], antioxidant [27], antigenotoxic [28], anticancer [19, 29–32], antimicrobial [33, 34], and neuroprotective [20, 35] properties, as well as the capability to modulate the hepatic lipid metabolism [36].

*Citrus bergamia *Risso et Poiteau, also called bergamot, is a less known fruit tree mainly growing in the southern coast of Calabria region (Italy), where the particular microclimate is ideal for its cultivation [37]. It is considered as hybrid between sour orange and lime (*Citrus aurantifolia* [Christm. & Panzer] Swingle) or between* Citrus aurantium* L. (sour orange) and* Citrus limon* L. Burm. f. (lemon) or a mutation of the latter [37]. Bergamot essential oil (BEO) is the main commercial product of bergamot industry, which take part in the composition of many fragrances [38]. Recently, BEO has been evaluated for its neuroprotective [39] and antiproliferative [40, 41] effects. Over the years, bergamot juice (BJ) has been considered a byproduct of BEO industry; however, within the last decade, it has gained attention by the scientific community due to its potential health effects. Indeed, very recently, its antioxidant [42, 43], anti-inflammatory [18, 44–47], anticancer [29–31, 48], and antihyperlipidemic effects [49, 50] have been reported. What is left of bergamot fruit after the extraction of BEO and its squeezing is called bergamot pastazzo to which we previously characterized the flavonoids and pectins content [7]. The flavonoid profile of the bergamot peel consisted of common* Citrus* species flavanone rutinosides and neohesperidosides derived from naringenin, eriodictyol, and hesperetin. Commercial pectinolytic and cellulolytic enzymes were able to hydrolyze the flavonoid glycosides and the pectic oligosaccharides present in bergamot peel [51]. We have also investigated the prebiotic potential of a pectic oligosaccharide-rich extract enzymatically derived from bergamot peel using pure and mixed cultures of human faecal bacteria [52]. A prebiotic is a nondigestible food ingredient able to modulate the microbiota composition of the large bowel, by selectively stimulating the beneficial bacteria through production of short chain fatty acids [53]. The results obtained demonstrated that bergamot oligosaccharides resulted in a high increase in the number of beneficial bacteria, such as bifidobacteria and lactobacilli, whereas the clostridial population decreased. The evaluation of the antimicrobial activity of flavonoids extracted from bergamot peel demonstrated that both the whole extract and the pure compounds neohesperidin, hesperetin (aglycone), neoeriocitrin, eriodictyol (aglycone), naringin, and naringenin (aglycone) were active against a range of Gram-negative bacteria, including* Escherichia coli*,* Pseudomonas putida*, and* Salmonella enterica* [7]. Studies on the biological activities of* Citrus bergamia* derivatives are summarised in Table 1.

## 3. *Helicobacter pylori* Infection

*Helicobacter pylori* is a microaerophilic Gram-negative bacterium able to colonize the human gastric mucosa. It has been identified as group 1 carcinogen by the International Agency for Research on Cancer and it is known to be associated with chronic gastritis, peptic ulcers, atrophic gastritis, intestinal metaplasia, and lymphoma or cancer development [54, 55]. Although infection rates are similar for men and women, they progressively increase with age. Approximately 50% of the world population is infected with* H. pylori*, with prevalence rates ranging from 20% to more than 80% in certain countries [56]. The estimated prevalence is 70% in developing countries, mainly in the young population, and 30%–40% in the developed countries [57]. Although the route of transmission of* H. pylori *remains unclear, the faecal-oral route represents an important pathway for its spread [58].

Amongst the genetic determinants causing* H. pylori* virulence, the cytotoxin-associated gene* (cagA)* and the vacuolating cytotoxin gene* (vacA)* play a crucial role.* VacA*, which is present in all* H. pylori* strains, contains at least two virulence parts [59], whereas* cagA*, not present in every* H. pylori* strain, is a marker for a pathogenicity island (PAI) [60]. In particular,* cagA* seems to be associated with more severe clinical outcomes and an increased risk of developing gastric cancer and peptic ulcer [61]. Type s1/m1 strains also produce a higher level of cytotoxin activity than other genotypes. A strong association between* cagA* and* vacA* signal sequence type s1 has been reported with strains carrying s1 m1 mosaic combination being able to secrete a vacuolating cytotoxin [56, 62]. A recent investigation on the impact of* H. pylori* antigens to the gastric mucosal barrier demonstrated a deleterious dose-influence effect of the lipopolysaccharide (LPS) [63]. The current treatment for* H. pylori* uses a combination of antimicrobial and antiacid agents [64]. A triple therapy consisting of a proton pump inhibitor (PPI) and two antibiotics, amoxicillin (AMX) and clarithromycin (CLA), or metronidazole (MTZ) and CLA and a quadruple therapy obtained by the association of PPI, bismuth, and two antibiotics (AMX + CLA or MTZ + tetracycline (TET)) are used [56]. Nevertheless, due to increasing antibiotic resistance, eradication rates were reduced to 70–80% over the last few years. Due to the different clinical outcomes of* H. pylori* infection in humans and to the increased number of side effects to common treatments, more effort is needed to find novel natural or synthetic anti-*H. pylori* agents. The high infection rate of* H. pylori* in low-income countries, together with the poor management in the application of antibiotics, has increased the need for finding novel anti-*H. pylori* agents from medicinal plants. As a result, several naturally occurring substances have been investigated as potential alternatives for the treatment of* H. pylori* infection [65–68]. For instance, we have recently demonstrated that polyphenolic fractions from almond skin were active against standard strains and clinical isolates of* H. pylori* [4]. A recent investigation reported the potential use of several medicinal herbs for the treatment of* H. pylori* and its complications [69]. Clinical trials are therefore warranted to test the efficacy of novel natural drugs against* H. pylori* infection.

## 4. Effect of* Citrus* Fruits and Their Derivatives against* Helicobacter pylori*

It is well established that diet plays a crucial role in gastric carcinogenesis and* Citrus* intake has been associated with a lower risk of stomach cancer [70, 71]. Out of approximately 870,000 noncardia gastric cancer cases, 74.7% have been attributed to* H. pylori* infection [72]. Therefore, a relevant number of studies have focused on the potential of* Citrus* fruits and their derivatives against* H. pylori* both in vitro and in vivo.

We have recently investigated the effect of bergamot juice against* H. pylori* in vitro and the potential therapeutic combination between bergamot juice and the antibiotics AMX, CLA, and MTZ [11]. We used two reference American Type Culture Collection strains of* H. pylori* (ATCC 43504 and ATCC 49503) and thirty-two clinical isolates recovered from gastric biopsy samples of dyspeptic adults undergoing digestive endoscopy. The 2.5% (v/v) concentration of BJ inhibited the growth of the clinical isolates tested by 50%, while 5% of BJ reduced* H. pylori* viability to 90%. Furthermore, concentration dependent killing was observed with BJ against all the tested strains. A >3log_10_ difference in colony-forming unit (CFU) between BJ 1.25% and BJ 20.0% with* H. pylori* ATCC 49503 was achieved after 2 h, whereas complete killing was achieved with BJ (10% and 20%) after 8 h and with BJ (5%) after 24 h. BJ gave less killing against* H. pylori* ATCC 43504, with a complete bacterial killing after 6 h exposure (20% BJ). This outcome could be explained by the increased antibiotic resistance of* H. pylori* ATCC 43504 compared to* H. pylori* ATCC 49503 [4]. In the combination assays, the association of BJ with the reference antibiotics determined a reduction of >6log_10_ for* H. pylori* ATCC 49503 after 8 h and for* H. pylori* ATCC 43504 after 24 h exposure to BJ. The association of BJ with the three reference antibiotics was more effective against* H. pylori* ATCC 49503 than* H. pylori* ATCC 43504. The most effective combination was BJ and CLA against the clinical isolate HP6 and HP61, with more rapid bacterial killing achieved with the cagA-positive strain (HP6).

This data confirmed the combination between BJ and the three reference antibiotics AMX, CLA, and MTZ had a synergistic effect against ATCC and clinical isolates of* H. pylori*. BJ was effective against* H. pylori* strains, both alone or in combination with antibiotics, and could therefore be used as novel strategy for the treatment of antibiotic resistance. Although the exact mechanism of action is not known, we believe that the synergistic effect may be due to the initial damage of the microbial lipid membrane by the plant compound, which would increase the permeability of the bacteria to the antibiotic.

Other authors have also reported on the activity of* Citrus* fruits and derivatives against* H. pylori*: two extracts (hexane and acetone) obtained from the leaves of* Citrus unshiu*,* Citrus sinensis*,* Citrus paradisi*, and* Laurus nobilis* demonstrated antimicrobial activity in vitro against a clinical isolate of* H. pylori*, with zone of inhibition diameters ranging within 0–30 mm and minimal inhibitory concentration (MIC) values of 1 : 512–1 : 4096 dilutions [73]. The antibacterial action of 30 Chinese herbal medicines which have been frequently used to treat gastritis-like disorders was tested against a standard and 5 clinical isolates of* H. pylori: Citrus reticulata* had MIC values close to 60 *μ*g/mL [74].

Rozza et al. [75] have reported on the gastroprotective mechanisms of* Citrus lemon* essential oil and its major compounds limonene and *β*-pinene and their activity in vitro against* H. pylori* ATCC 43504: results showed a gastroprotective effect of* Citrus lemon* and limonene with MIC values of 125 and 75 *μ*g/mL, respectively. The role of *β*-myrcene, a minor constituent of essential oil from* Citrus aurantium*, in preventing peptic ulcer disease has been evaluated: results showed *β*-mircene was an effective inhibitor of gastric and duodenal ulcers through an increase in the levels of gastric mucosa defence factors [76]. Hesperetin-7-*O*-glucoside, produced by fungal enzymatic conversion of* Citrus* hesperidin, was active in vitro against* H. pylori*, also inhibiting human intestinal maltase and HMG-CoA reductase [77]. Other biologically active derivatives from* Citrus* fruits, such as 4′-geranyloxyferulic acid and boropinic acid, as well as sudachitin and 3′-demethoxysudachitin, were found active against* H. pylori* in vitro [78, 79].

In vivo studies on the effect of auraptene on* H. pylori* colonization of the gastric mucosa showed that a dietary supplementation with 50 ppm of auraptene suppressed* H. pylori* colonization, although it did not reduce gastric inflammation [80]. The attenuation of gastritis was obtained by a reduction of* H. pylori* colonization, expression of CD74, and production of proinflammatory mediators in mice [81, 82]. The same authors identified bergamottin as one of the best candidates for the treatment of* H. pylori* infection [83].

A number of studies have investigated the effect of plant extracts against urease activity [84, 85]. It is believed that the urease secreted by* H. pylori* is a crucial enzyme able to protect the bacteria within the acidic environment of the human stomach. The Iranian* Citrus aurantifolia* and* Citrus aurantium *extracts revealed a strong urease inhibitory activity, with IC_50_ values of 432 and 465 *μ*g/mL [84, 85].

Even though the data presented indicate that* Citrus* fruits and their bioactive compounds could represent appropriate sources for the development of novel effective compounds against* H. pylori*, additional in vivo toxicological evaluations are warranted. Intervention studies should also take into account the fact that the pharmaceutical doses supporting a protective effect may derive from a synergistic action of the numerous bioactive compounds present in a plant extract. Tables 2 and 3 summarised the anti-*H. pylori* activity of* Citrus* fruits derivatives and their compounds.

## 5. Conclusion

The renewed interest in exploring natural sources of alternative anti-*H. pylori* agents has been forced by the increased side effects to common treatments and the spread of antibiotic resistance. The use of plant extracts could help control* H. pylori* infection in low-income countries, where the rate of infection is particularly high. However, more clinical investigations evaluating the toxicity, chemical or physical stability, and bioavailability of plant bioactives are warranted. Several studies have evaluated the effectiveness of* Citrus* fruits derivatives and their bioactive compounds against* H. pylori*, gastric carcinoma, and urease activity, suggesting that, either alone or in combination with antibiotics, they could represent useful sources to help eradicate* H. pylori* and avoid gastric ulcer relapse.

## Figures and Tables

**Table 1:** Studies employing *Citrus bergamia* derivatives and their experimental model.

*Citrus bergamia* derivatives	Study type	Experimental model	Reference
Flavonoid-rich fractions from peel	In vitro	Bacteria and yeasts	[7]
Essential oil	In vitro	Mycoplasma	[10]
Juice	In vitro	SH-SY5Y cells	[29]
Juice	In vivo	SK-N-SH/LAN-1xenograft mice	[30]
Juice	In vitro	HepG2 cells	[31]
Essential oil	In vitro	NMDA-exposed SH-SY5Y cells	[39]
Liposomal essential oil	In vitro	SH-SY5Y cells	[40]
Essential oil	In vitro	SH-SY5Y cells	[41]
Flavonoid-rich fractions from juice	In vitro	H_2_O_2_-exposed A549 cells	[42]
Flavonoid-rich fractions from juice	In vitro	Fe^3+^-exposed A549 cells	[43]
Flavonoid-rich fractions from juice	In vitro	LPS-stimulated THP-1 cells	[44]
Flavonoid-rich fractions from juice	In vivo	DNBS-injected mice	[45]
Flavonoid-rich fractions from juice	In vivo	Intestinal ischemia/reperfusion injury	[46]
Flavonoid-rich fractions from juice	In vitro	*β*-Amyloid-stimulated THP-1 cells	[47]
Flavonoid-rich fractions from juice	In vitro	HT-29 cells	[48]
Juice extract	Human	Subjects with moderate hypercholesterolemia	[50]
Flavonoids and pectic oligosaccharides from peel	In vitro	Solubilization with commercial enzyme preparations	[51]
Pectic oligosaccharide-rich extract from peel	In vitro	Colonic model fermentation	[52]

**Table 2:** Studies investigating the anti-*Helicobacter pylori* activity of *Citrus* fruits.

*Citrus* species	Strain	MIC	Reference
*C. bergamia*	ATCC 43504	0.625–5.0 *µ*g/mL	[11]
	ATCC 49503		
	32 clinical isolates		
*C. unshiu*	Clinical isolates	1 : 512–1 : 4096 dilutions	[73]
*C. sinensis*			
*C. paradisi*			
*C. reticulata*	5 clinical isolates	60 *µ*g/mL	[74]
*C. lemon*	ATCC 43504	125 *µ*g/mL	[75]
*C. aurantifolia*	Urease inhibitory activity	432 *µ*g/mL	[84]
*C. aurantium*	Urease inhibitory activity	465 *µ*g/mL	[85]

**Table 3:** Compounds extracted from *Citrus *fruits exerting anti-*Helicobacter pylori* activity.

Compound	Reference
*β*-Myrcene	[76]
Hesperetin-7-O-glucoside	[77]
Boropinic acid	[78]
Sudachitin 3′-demethoxysudachitin	[79]
Auraptene	[80]
Auraptene	[81]
Auraptene	[82]
Bergamottin	[83]

## Abbreviations

BEO: Bergamot essential oil
BJ: Bergamot juice
*cagA*: Cytotoxin-associated gene
*vacA*: Vacuolating cytotoxin gene
PAI: Pathogenicity island
LPS: Lipopolysaccharide
PPI: Proton pump inhibitor
AMX: Amoxicillin
CLA: Clarithromycin
MTZ: Metronidazole
TET: Tetracycline
MIC: Minimal inhibitory concentration
IC_50_: Half maximal inhibitory concentration.
